# Activation of G protein-coupled receptors by ketone bodies: Clinical implication of the ketogenic diet in metabolic disorders

**DOI:** 10.3389/fendo.2022.972890

**Published:** 2022-10-20

**Authors:** Valentina Spigoni, Gloria Cinquegrani, Nicolas Thomas Iannozzi, Giulia Frigeri, Giulia Maggiolo, Marta Maggi, Vanessa Parello, Alessandra Dei Cas

**Affiliations:** ^1^ Endocrinology and Metabolic Diseases, Department of Medicine and Surgery, University of Parma, Parma, Italy; ^2^ Division of Nutritional and Metabolic Sciences, Azienda Ospedaliero-Universitaria di Parma, Parma, Italy

**Keywords:** ketogenic diet, ketone bodies, GPCR (G protein coupled receptors), metabolic disorder, very low carbohydrate ketogenic diet

## Abstract

Ketogenesis takes place in hepatocyte mitochondria where acetyl-CoA derived from fatty acid catabolism is converted to ketone bodies (KB), namely β-hydroxybutyrate (β-OHB), acetoacetate and acetone. KB represent important alternative energy sources under metabolic stress conditions. Ketogenic diets (KDs) are low-carbohydrate, fat-rich eating strategies which have been widely proposed as valid nutritional interventions in several metabolic disorders due to its substantial efficacy in weight loss achievement. Carbohydrate restriction during KD forces the use of FFA, which are subsequently transformed into KB in hepatocytes to provide energy, leading to a significant increase in ketone levels known as “nutritional ketosis”. The recent discovery of KB as ligands of G protein-coupled receptors (GPCR) - cellular transducers implicated in a wide range of body functions - has aroused a great interest in understanding whether some of the clinical effects associated to KD consumption might be mediated by the ketone/GPCR axis. Specifically, anti-inflammatory effects associated to KD regimen are presumably due to GPR109A-mediated inhibition of NLRP3 inflammasome by β-OHB, whilst lipid profile amelioration by KDs could be ascribed to the actions of acetoacetate *via* GPR43 and of β-OHB *via* GPR109A on lipolysis. Thus, this review will focus on the effects of KD-induced nutritional ketosis potentially mediated by specific GPCRs in metabolic and endocrinological disorders. To discriminate the effects of ketone bodies *per se*, independently of weight loss, only studies comparing ketogenic *vs* isocaloric non-ketogenic diets will be considered as well as short-term tolerability and safety of KDs.

## Introduction

### Metabolism of ketone bodies

**Figure 1:**
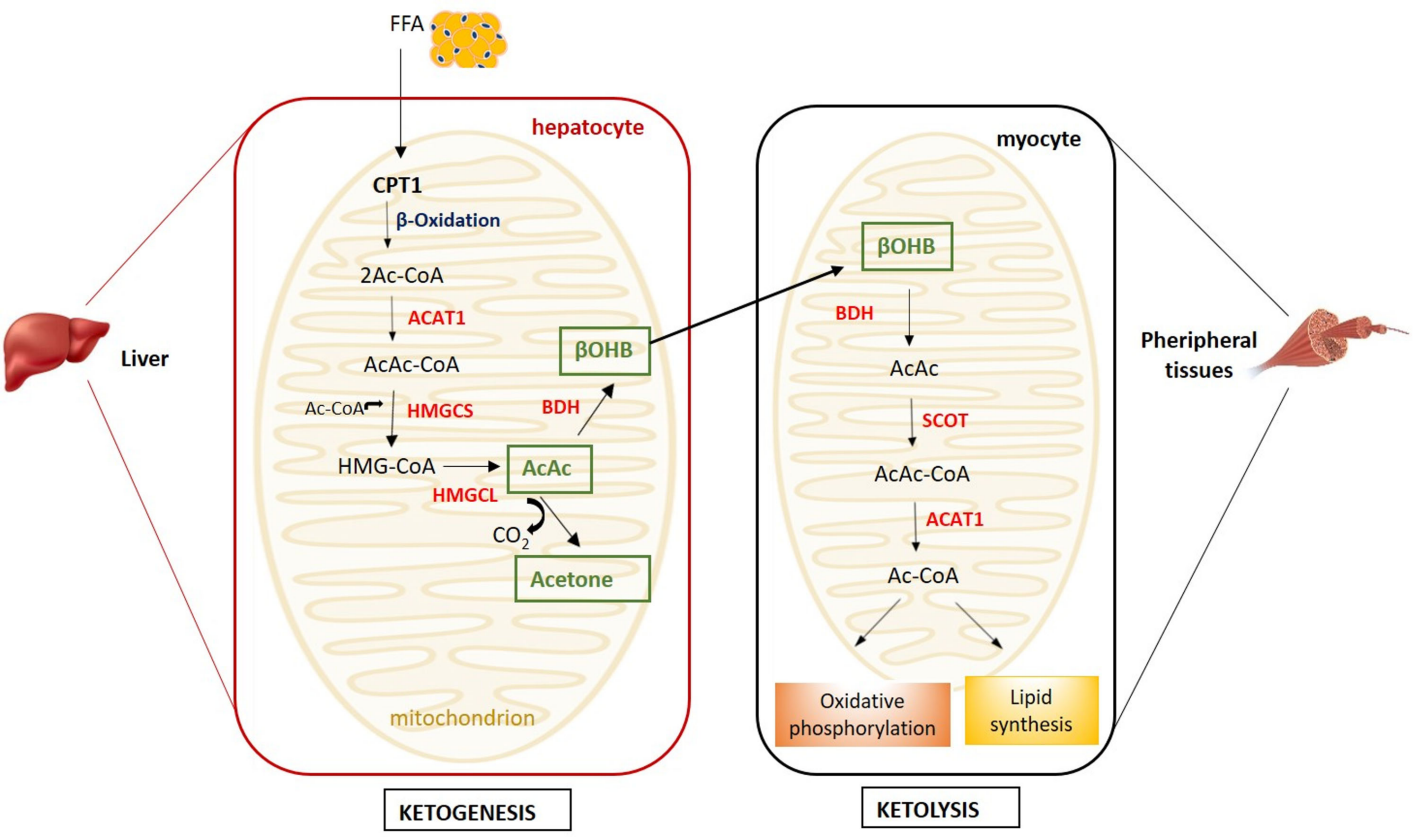
Metabolism of ketone bodies. Ketogenesis takes place in hepatocyte mitochondria, while ketolysis in the mitochondria of peripheral tissues (i.e. muscle, heart, kidney and brain). (ACAT1, acetyl coenzyme A acetyltransferase; AcAc, acetoacetate; Ac-CoA, acetyl-CoA; AcAc-CoA, acetoacetyl-CoA; BDH, β-hydroxybutyrate dehydrogenase; βOHB, β-hydroxybutyrate; CPT1, carnitine palmitoyltransferase 1; FFA, free fatty acids; HMGCL, HMG-CoA lyase; HMG-CoA, β-hydroxy-β-methylglutaryl-CoA; HMGCS, HMG-CoA synthase; SCOT, succinyl-CoA-3-ketoacid-CoA transferase).

Ketone bodies (KB) - β-hydroxybutyrate (β-OHB), acetoacetate and the less abundant acetone - are produced by ketogenesis in the hepatocyte mitochondria and converted into energy in the mitochondria of several extrahepatic organs (i.e. brain, heart, kidney cortex and skeletal muscle) by ketolysis (**Figure 1**) (1).

Briefly, in the ketogenesis process, free fatty acids (FFA) mobilised from the adipose tissue are transported into the liver mitochondria by carnitine palmitoyltransferase (CPT1) and undergo β-oxidation resulting into two acetyl-CoA molecules subsequently condensed to acetoacetyl-CoA by acetyl coenzyme A acetyltransferase (ACAT1) (2). The condensation of acetoacetyl-CoA with a third molecule of acetyl-CoA, by β-hydroxy-β-methylglutaryl-CoA (HMG-CoA) synthase, leads to the formation of HMG-CoA, an intermediate in the mevalonate and ketogenesis pathways and a precursor of cholesterol biosynthesis (3). The HMG-CoA lyase converts the HMG-CoA to the β-keto acid acetoacetate, which, in turn, can be converted to acetone and CO_2_ by non-enzymatic decarboxylation or to β-OHB by β-OHB-dehydrogenase. The liver releases β-OHB, which, in a process named ketolysis, is re-converted to acetoacetate and subsequently to acetyl-CoA by the succinyl-CoA-3-ketoacid-CoA transferase (SCOT), which is expressed in all mammalian cells with mitochondria, with the exception of hepatocytes (2) (**Figure 1**).

Acetyl-CoA enters the Krebs cycle for ATP production through oxidative phosphorylation.

KB utilization as metabolic substrate is energetically more efficient in terms of ATP synthesis per molecule of oxygen invested than glucose or FFA and allows the transfer of lipid-derived energy from liver to extrahepatic organs as an alternative fuel source (4). Alternatively, acetyl-CoA deviates from the Krebs cycle to lipid synthesis. Acetone does not convert backwards to acetyl-CoA and it is excreted through urine or exhaled (5).

### Conditions associated to physiological ketosis

Several conditions are associated to physiological plasma ketosis. KB plasma levels are normally < 0.5mM, but during fasting, a decrease in insulin/glucagon ratio is paralleled by a concomitant increase in plasma FFA and KB, which, in the presence of a prolonged starvation, may excessively increase up to 5-fold (1.3 mM) and 20-fold (7-10 mM), respectively (6–8). Prolonged exercise may also induce a physiological increase in plasma KB up to 1-2 mM (1). Other biological conditions, such as pregnancy or the neonatal period, are associated to a mild increase in ketosis (1).

The nutritional ketosis following a ketogenic diet (KD) regimen also represents a physiological mechanism, in which KB concentrations are < 3.0 mM and blood pH remains within the normal range (1, 9). Conversely, diabetic ketoacidosis, a severe acute complication of diabetes, is a pathological condition in which KB reach extremely high plasma concentrations (≥ 3.0 mM (10)) with a concomitant blood pH fall below the normal range (11). In diabetic conditions, insulin deficiency prompts the body to metabolize triglycerides and amino acids, instead of glucose, for energy. Serum levels of glycerol and FFA consequently rise due to exacerbated lipolysis (1). Moreover, glycerol provides the substrates for hepatic gluconeogenesis, which is further stimulated by the excess glucagon that accompanies insulin deficiency (1). In non-diabetic individuals, ketoacidosis is prevented as the preserved insulin secretion reduces the release of FFA from the adipose tissue, leading to a decreased ketogenesis in hepatocytes, accompanied by urinary KB excretion (9).

Thus, KB can be either a necessary nutrient or the reflection of a pathological status depending on their plasmatic concentrations.

## Definition and types of ketogenic diets

KDs were first used since the 1920s to treat refractory epilepsy in children (12) and increasing evidence shows encouraging results in pathological conditions such as Alzheimer disease (13), cancer (14), and metabolic diseases (15). KDs are defined as low-carbohydrate (usually to < 50 g/day) and adequate protein intakes to induce ketosis without restricting fat intake (16). Similar to fasting conditions, during a carbohydrate-restricted diet, the body undergoes metabolic changes to provide an energy source relying on gluconeogenesis and ketogenesis. Following depletion of glycogen stores, the body is forced to use FFA, which are subsequently transformed into KB in hepatocytes, to provide energy (1). KB become the primary energy source by cells with mitochondria and, most importantly, by the brain. This significant rise in KB in course of KD it is known as “nutritional ketosis” (17), a condition in which plasma KB concentration physiologically increases (at least > 0.5 mM (18)), resulting in a rather safe nutritional approach (19).

Different variants of the KD have been defined in the last few years, although a commonly accepted classification is still lacking.

Types of KDs includes: the classic long-chain triglycerides (LCT) KD, which is the most used KD, and it contains four times as many grams of fat for every 1 g of protein and carbohydrates (4:1 ratio) (20, 21). The medium-chain triglyceride (MCT, C6–C12) KD is more ketogenic than (LCT KD); MCTs are readily absorbed into the portal vein -as they do not require micelle formation with bile acids- and are directly transformed into KB. The MCT KD allows the intake of a higher ratio of carbohydrates and protein compared to the high-fat KD with consequent increase in adherence and prevention of muscle catabolism (22). Differently from high fat KD, the MCT KD is built only on the percentage of calorie from MCT oil (23). MCT KD is commonly used for the treatment of refractory childhood epilepsy (24, 25) and other neurological disorders such as cognitive dysfunction in Alzheimer’s disease and mild cognitive impairment (26).

The very low carbohydrate ketogenic diet (VLCKD) represents the most widespread KD type and is traditionally characterized by carbohydrate limitation (<50 g per day) with unrestricted fat consumption and a moderate increase in protein intake (0.8-1.2 g per day) (27). The total amount of calories to be provided for a single individual is based on anthropometric measurements, dietary intake and physical activity (28).

The very low calorie KDs have an extremely low caloric intake (400–800 kcal/day) and contain an amount of protein of 1.2–1.4 g/kg of ideal body weight, a very limited carbohydrate content (< 30–50 g/day, < 5–10%) and a determined amount of fats (∼20 g/day) (29).

Beyond calorie restriction, the increase in KB levels is associated with a specific anorexic effect, by preventing the increase in ghrelin secretion in response to weight loss, decreasing hunger (30). However, the exact threshold of ketosis needed to induce appetite suppression, as well as the exact mechanisms mediating such an effect, have yet to be elucidated (31). The suppression/blunting of hunger sensation and consequent reduction in caloric intake has a fundamental role in diet compliance and short-term weigh loss outcomes (32–34).

Generally, KDs are recommended for a limited time and the shift to a standard diet should be gradual and supervised (35). KDs may be fraught with side effects which usually disappear within a few days to a few weeks like nausea, vomiting, headache, tiredness, insomnia and exercise intolerance. Long-term adverse effects may include hepatic steatosis, hypoproteinemia and kidney stones (35). The main contraindications to implement a KD are pancreatitis, liver failure, primary carnitine deficiency, carnitine palmitoyltransferase deficiency, carnitine translocase deficiency, porphyrias, or pyruvate kinase deficiency (36).

It has recently emerged that KDs may hold additional beneficial effects on metabolism and cardiovascular risk reduction, which may be partially independent of weight loss. The increase in KB following a keto diet regimen would potentially produce tissue specific effects by the activation of cell membrane G protein-coupled receptors (GPCRs) which have been recently discovered to be engaged by β-OHB and acetoacetate.

## G protein-coupled receptors (GPCRs)

GPCRs - which represent the largest protein family in the human proteome - are signal transducers located in the plasma membrane of eukaryotic cells. GPCRs share a common structure consisting of seven 25-amino acid α-helical segments crossing through the plasma membrane, with the amino-terminus and carboxy-terminus outside the cell and in the cytoplasm, respectively (37). The variety of ligands capable of binding to GPCRs is notable, ranging from a single photon to large proteins and including ions, odorants, nucleotides, peptides, amines, lipids and metabolic intermediates. Following ligand engagement, GPCRs interact with G proteins in the plasma membrane leading to the production of cAMP and of other second messengers. A subsequent cascade of phosphorylation, involving extracellular regulated protein kinases 1/2 (ERK1/2), transduces cellular responses by modulating cell metabolism and function (38).

Over time, the number of GPCRs discovered has increased as well as that of their binding energy substrates such as fatty acids and sucrose, and key metabolic intermediates including acetate and lactate (39–41). The recent discovery of KB as GPCR ligands has aroused a great interest in understanding whether some of the clinical effects associated to KDs might be mediated by ketone/GPCR axis; however, data are scarce in the literature.

Thus, this review will focus on the effects of KD-induced nutritional ketosis potentially mediated by specific GPCRs in metabolic and endocrinological disorders.

## β-hydroxybutyrate reduces atherosclerosis and inflammation by engaging GPR109A

β-OHB is the most represented KB in humans and its concentrations typically reach ∼0.2–0.4 mM after an overnight fast, ∼1–2 mM following 2–3 days of fasting, and plateaus at ∼6–8 mM upon prolonged starvation (1, 42–44).

Most of β-OHB actions seem to be mediated by the activation of a specific GPCR, namely GPR109A, also known as Hydroxycarboxylic Acid Receptor 2 (HCAR_2_). GPR109A is classically recognized as the receptor for butyrate and nicotinic acid and is abundantly expressed in adipocytes, macrophages, and neutrophils (45). The plasma concentrations of β-OHB able to engage GPR109A (EC_50 =_ 0.77 ± 0.06 mM) (46) are those achievable after ∼2–3 days of starvation (1), or following a ketogenic diet regimen (47–49).

The binding of β-OHB to GPR109A is implicated in the reduction of both lipid metabolism and inflammation (**Table 1**). In adipocytes, GPR109A activation results in Gi/G0 protein-mediated inhibition of adenylate cyclase, leading to a mitigated cAMP response with a consequent reduction in hormone sensitive lipase activity, resulting in lipolysis inhibition (57). The consequent reduced FFA release and hepatic delivery limit substrate availability for hepatic triglyceride and has been proposed as a negative feedback mechanism in KB synthesis (46, 58). Accordingly, a calorie-restricted diet or β-OHB administration resulted in the suppression of hepatic lipid accumulation through GPR109A/AMP-activated protein kinase (AMPK) pathway in aged rats (51). If the results on lipolysis inhibition are robust, less consistent are the findings on the resulting modifications in plasma lipoprotein profile (59).

**Table 1:** Summary of studies on GPCR-mediated actions of ketone bodies.

Reference	Animal or cell model	Ketone body (concentration)	GPCR involved	Effects
Zhang SJ 2021(50)	BMDMs from wt mice	β-OHB (10 mM)	GPR109A	↓ M1 polarization ↑ Promoting cholesterol efflux in macrophages
Lee AK 2020 (51)	Aged rats	β-OHB (10 and 100 mg/kg/day)	GPR109A	↑ AMPK levels ↓ Hepatic ER stress and lipid accumulation
	HepG2 cells	β-OHB (200 and 400 µM)		
Rahman M 2014 (45)	KD-fed mice (wt and GPR109A^-/-^)	β-OHB	GPR109A	↑ COX1-mediated neuroprotective phenotype of monocytes and/or macrophages
Fu SP 2015 (52)	Primary rat microglial cells	β-OHB (1.5 mM)	GPR109A	↓ LPS-induced NF-κB activation and IL-1β, IL-6 and TNF-α production
Chen Y 2018 (53)	C57BL/6 mice	β-OHB (3 mmol/kg)	GPR109A	↑ M2 phenotype of intrahepatic macrophages
Won YJ 2013 (54)	Superior cervical sympathetic ganglionic neurons	β-OHB (10 mM)	GPR41	↓ Ca^2+^ channel currents
Kimura I 2011 (55)	HEK293 expressing mouse GPR41 and sympathetic neurons	β-OHB (0.1 and 1 mM)	GPR41	↓ propionate-induced ERK1/2 activation ↓ activity in sympathetic neurons
Miyamoto J 2019 (56)	GPR43-overexpressing HEK293	Acetoacetate (0.3 mM)	GPR43	↓ cAMP levels induced by forskolin
	*Gpr43^−/−^* mice	Acetoacetate (500 mg/kg)		↑ lipolysis by activating plasma LPL activity

BMDM, bone marrow-derived macrophages; β-OHB, β-hydroxybutyrate; AMPK, AMP-activated protein kinase; COX1, cyclooxygenase 1;ERK1/2, Extracellular signal-regulated protein kinase; LPS, lipopolysaccharide; NF-κB, nuclear factor k B. ↓, decrease; ↑ increase.

In addition to the effects on lipolysis inhibition, emerging evidence points to GPR109A as the molecular transducer of β-OHB-mediated effects also on the mitigation of the inflammatory burden, through the reduction in NLR family pyrin domain containing 3 (NLRP3) inflammasome activity (50). Briefly, the inflammasome is a multiprotein intracellular complex which can be activated by diverse signals such as endoplasmic reticulum (ER) stress, reactive oxygen species (ROS), and excessive Ca^2+^ leading to caspase 1 activation which in turn activates proinflammatory interleukin (IL)-1β and IL-18. Specifically, in macrophages, inflammasome activation triggers pro-atherogenic M1 polarization -in contrast to anti-inflammatory M2- contributing to the pro-inflammatory e pro-atherogenic environment (60).

A recent work demonstrated that the exogenous administration of 200 mg/kg/day of β-OHB resulted in a strong fat reduction in *apoE ^−/−^* atherosclerotic mice even in the presence of a high‐fat diet. β-OHB decreased pro-inflammatory M1 polarization and cholesterol accumulation in bone marrow‐derived macrophages (BMDMs) (50). These protective effects were no longer evident in GPR109A ^−/−^ and NLRP3 ^−/−^ BMDMs, indicating that β-OHB attenuates atherosclerosis *via* GPR109A-NLRP3 dependent pathway. Mechanistically, β-OHB - *via* GPR109A - promoted the influx of Ca^2+^ by reducing Ca^2+^ release from ER thereby maintaining Ca^2+^ storage and inhibiting its depletion induced by NLRP3 inflammasome activation. Overall, this study suggests that the treatment with β-OHB significantly blunts systemic inflammation and atherogenesis mostly through GPR109A-expressing macrophages (50).

Most of the knowledge concerning the anti-inflammatory action of β-OHB mediated by GPR109A comes primarily from studied conducted in neurodegenerative diseases. Nicotinic acid, a GPR109A agonist, induced a neuroprotective phenotype of monocytes/macrophages in a mouse model of stroke resulting in the reduction of ischemic brain damage (45). GPR109A activation in macrophages led to Prostaglandin D2 (PGD2) production by Cyclooxygenase (COX)-1 and PGD2 synthase, underscoring the key role of PGD2 in the regulation of inflammation and innate immune response (61).

Another study demonstrated that β-OHB, inhibited pro-inflammatory enzyme and cytokine [tumor necrosis factor (TNF)-α, IL-1β, and IL-6] production in microglia *via* GPR109A, through the reduction of nuclear factor-κB (NF-κB) activation (52). This anti-inflammatory action of β-OHB has been recently proposed as a tool to prevent (or slow down) also the progression of Parkinson’s disease.

**Figure 2:**
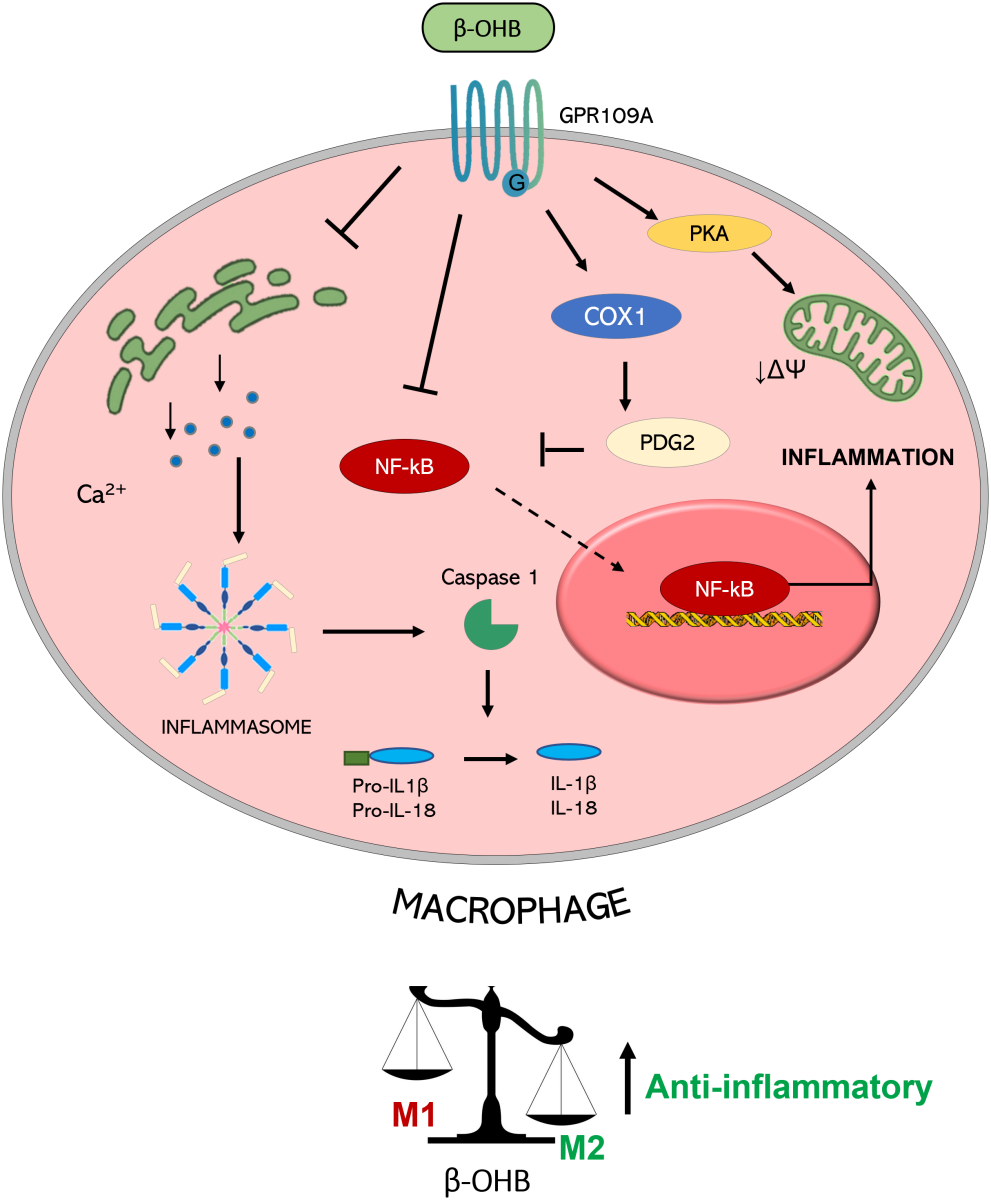
Molecular mechanisms triggered by GPR109A-β-OHB axis in macrophages. GPR109A engagement by β-OHB leads to a reduced inflammasome activation mediated by a reduction of Ca^2+^ release from endoplasmic reticulum. This inhibition of inflammasome activity causes a lower IL-1β and IL-18 release from macrophages. A reduction of inflammatory mediator release is also achieved by the inhibition of the transcription factor NF-κB which can be mediated by the COX-1-mediated synthesis of PDG2, a known inhibitor of NF-κB activity. All together these effects converge in the induction of an anti-inflammatory M2 phenotype of macrophages. This switch is also obtained by a PKA-mediated decrease in the mitochondrial membrane potential which guides M2 polarization. (PGD2: Prostaglandin D2; COX-1: Cyclooxygenase 1; PKA: Protein kinase A; NF-κB: nuclear factor-κB; IL: interleukin; βOHB: β-hydroxybutyrate; ΔΨ: mitochondrial membrane potential).

GPR109A-mediated anti-inflammatory action of β-OHB was shown to be also protective in alcohol hepatitis, characterized by liver inflammation which may progress into systemic inflammatory response and high neutrophil infiltration in the liver (62). The β-OHB intake in mice has an anti-inflammatory and hepatoprotective role through a GPR109A-dependent pathway. Specifically, β-OHB supplementation (i.p. 3 mmol/kg) induced anti-inflammatory IL-10 transcripts and promoted the switch of mouse intrahepatic macrophages towards an anti-inflammatory M2 phenotype (53). This effect was mediated by the activation of protein kinase A (PKA), which reduces mitochondrial membrane potential and was no longer evident in GPR109A-knockout mice (63). GPR109A-mediated effects of β-OHB in modulating macrophage polarization (shown in **Figure 2**) support a key role of GPR109A signalling as anti-inflammatory regulator of the low-grade inflammation associated with insulin resistance and obesity (64).

## β-hydroxybutyrate might modulate cell metabolism by engaging GPR41

Some evidence (**Table 1**) points to β-OHB as a novel ligand also for GPR41, a GPCR expressed in human adipocytes (65), sympathetic neurons (54) colon epithelial, endothelial (66) and mononu clear cells, and classically activated by short-chain fatty acids (SCFAs) such as acetate, propionate, and butyrate  (67). Studies on knockout mice implicate GPR41 in chronic inflammatory disorders such as obesity, colitis, asthma and arthritis, although its protective or causative role is inconsistent among studies (68). Propionate potently activated sympathetic nervous system (SNS) and β-OHB - through unclear molecular mechanisms - antagonized SCFA-GPR41 signaling thereby suppressing SNS activity (55), which is known to be finely regulated for the balance of energy intake, dietary excess and starvation. This β-OHB inhibitory effect on SNS was demonstrated both in cell culture stimulated with β-OHB 10-100 mM and in streptozotocin-induced diabetic mice, in which β-OHB concentration reached 2.6 mM (55). Although other studies consider β-OHB a GPR41 agonist, rather than antagonist (54), it is established that β-OHB can control energy balance by directly regulating GPR41-mediated sympathetic activation (55). Further studies are necessary to confirm these findings and to unravel the precise molecular mechanism underlying the involvement of GPR41 in the modulation of cell metabolism.

## Acetoacetate is an endogenous ligand of GPR43

GPR43 is a SCFA receptor which reduces cAMP levels, activates the ERK1/2 pathway, and increases Ca^2+^ concentrations (69). Although highly expressed in adipose tissues, it is also involved in the modulation of inflammation and body metabolism through gut microbiota (70, 71).

Recent evidence reported that the KB acetoacetate functions as an endogenous activator of GPR43 with binding affinity comparable to that of SCFA (i.e. propionate and acetate) under ketogenic conditions (**Table 1**). As already stated, during fasting KB plasma concentrations were increased (>0.5 mM) and SCFA levels were highly decreased (from 0.5 to 0.1 mM), suggesting that the primary ligand for GPR43 in plasma switches from SCFAs to KB under fasting conditions.

Under ketogenic conditions acetoacetate increased plasma lipid utilization by controlling the activation of lipoprotein lipase (LPL) *via* adipose GPR43 (56). Furthermore, plasma levels of glucose and triglycerides were significantly reduced, and cholesterol levels increased, in wild-type compared to *Gpr43^−/−^* mice. Similarly, the decrease in body weight was significantly blunted in the *Gpr43^−/−^* mice compared with the wild-type mice during fasting. Thus, the acetoacetate-GPR43 axis plays a key role on metabolic homeostasis by controlling body weight and lipid levels.

## Effects of KDs in endocrinological and metabolic disorders

The following paragraphs will discuss the results of clinical trials on the effects of KD in different metabolic and endocrinological disorders [obesity, diabetes, non-alcoholic fatty liver disease (NAFLD) and polycystic ovary syndrome (PCOS)]. The rationale for the use of KDs is the substantial efficacy in short-term weight loss; however, it is still unclear whether KDs may have additional benefits *vs* isocaloric non-ketogenic diets. In this context we will explore the hypothesis that KB, by binding to GPCR family receptors, may mediate some of these additional beneficial effects (i.e lipid metabolism and anti-inflammatory actions). To discriminate the effects of KB *per se*, independently of weight loss, only studies comparing ketogenic *vs* isocaloric non-ketogenic diets have been herein considered, unless otherwise specified. Short-term tolerability and safety of KDs are reported in **Tables 2** and **3**.

**Table 2:** Summary of trials evaluating KD effects vs comparative diet in obese subjects.

Reference	Sample description	Duration	Intervention	Ketone test (concentration in KD group)	Beneficial effects of the KD *vs* control diet	Side effects of the KD
Sánchez E 2021 (72)	n=30 patients with moderate obesity	6 months	VLCKD *vs* hypocaloric Mediterranean Diet	Not tested	↓BMI ↓total body fat ↓soluble ICAM-1	- Gastrointestinal disorders (n=3) - non-fatal episode of heart failure (n=1)
Falkenhain K 2021 (73)	n=155 overweight/obese subjects	12 weeks	Mediterranean ketogenic diet *vs* calorie-restricted, low-fat diet	Breath acetone	↓Weight loss ↓HbA1c	No adverse events reported
Michalczyk MM 2020 (48)	n=100 hyperinsulinemic overweight/obese females	12 weeks	LCKD *vs* isocaloric control diet	β-OHB= 3.46±0.06 mM	↓ Glucose ↓ Insulin ↓ HbA1c ↓ HOMA-IR ↓ TG ↑ HDL-C ↓BMI ↓ waist circ. ↓hip circ.	No adverse events reported
Perissiou M 2020 (74)	n=64 obese subjects	8 weeks	LC *vs* standard dietary advice (both + exercise program)	Ketosis defined as β-OHB >0.3mM	↓ body fat ↓ visceral adipose tissue ↓ fat mass ↓ muscle mass ↔ lipids ↓C-reactive protein	↓lean muscle mass
Sun S 2019 (75)	n=58 overweight females	4 weeks	LC *vs* control diet	Urinary keto-stick	↓ BMI ↓waist circ. ↔ fasting glucose ↔ lipids	↑ fatigue ↑ constipation ↑ diarrhea ↑ headache
Hall KD 2016 (49)	n=17 overweight/obese males	4 weeks of control diet followed by 4 weeks of intervention diet	LCKD *vs* isocaloric control diet	Plasma acetoacetate= 0.78±0.07 mM β-OHB=0.76±0.07 mM	↓ energy expenditure ↓insulin ↓C-peptide ↑ FFA ↓TG ↓ leptin	No adverse events reported
Merra G 2016 (76)	n=25 overweight/obese subjects	3 weeks	VLCKD *vs* VLCD	Urinary keto-stick	↓ body weight ↓waist circ. ↔ lean bodymass	No adverse events reported
Lobley GE 2014 (33)	n=12 obese males	4 weeks per treatment	*Cross-over*LC *vs* MCD	Plasma acetoacetate= 0.40 mMβ-OHB= 0.66 mM	↓ hunger score ↔ brain glucose uptake	No adverse events reported
Johnstone AM 2008 (34)	n=17 obese males	4 weeks per treatment	*Cross-over*high-protein, LCKD *vs* high-protein, MCD	Plasma β-OHB= 1.52 mM	↓ hunger ↓ Ad libitum energy intake ↑ weight loss	No adverse events reported
White AM 2007 (47)	n=19 overweight/obese subjects	2 weeks	LCKD *vs* non-ketogenic LC	Plasma β-OHB= 0.72±0.18 mM	↔weight loss ↔fat mass Positive correlation between ketones and perceived exertion	↑ fatigue ↑ total mood disturbance
Choi HR 2018 (32)	n=46 obese subjects	2 weeks	ketogenic nutrition drink *vs* isocaloric balanced nutrition drink	Urinaryketo-stick	↔ weight loss ↔ fat mass ↑ HDL chol.	↑ nausea ↑ constipation
Yancy WS Jr 2004 (77)	n=120 overweight, hyperlipidemic subjects	24 weeks	LCKD*vs*low fat diet	Urinary Ketones (semi-quantitative)	↑ weight loss ↓ TG ↔LDL chol. ↑HDL chol.	↑ headache ↑ halitosis ↑ constipation ↑ muscle cramps ↑ diarrhea ↑ weakness ↑ rash
Vazquez JA 1994 (78)	n=16 severely obese females	28 days	ketogenic vs non-ketogenic VLCD	Plasma β-OHB= 3.1±0.5 mM	↓ plasma glucose ↑ protein oxidation ↔ lipolysis	No adverse events reported
Albanese A 2019 (79)	n=178 obese subjects (candidate to bariatric surgery)	3 weeks	VLCKD *vs* VLCD	Not tested	↑ weight loss ↓ post-operative hospital stay	No adverse events reported
Schiavo L 2021 (80)	n=48 obese subjects undergoing gastric balloon procedure	4 months	Low-calorie ketogenic diet *vs* low-calorie diet	Ketonemia=0.18±0.2 mM	↓ decrease in fat-free mass and resting metabolic rate ↑ fat mass decrease	No adverse events reported

LCKD, low carbohydrate ketogenic diet; VLCKD, very low carbohydrate ketogenic diet; VLCD, very low calorie diet; KD, ketogenic diet; MCD, moderate carbohydrate diet; LC, low carbohydrates; BMI, body mass index; β-OHB, 3-hydroxy butyrate; TG, triglycerides. ↓, decrease; ↑, increase; ↔ no changes.

**Table 3:** Summary of trials evaluating KD effects vs comparative diet in subjects with type 2 diabetes.

Reference	Sample description	Duration	Intervention	Ketone test (concentration in KD group)	Beneficial effects	Side effects of the KD
Hussain TA 2012 (81)	n=363 with T2D= 102 Age= 37.2±0.4 yr BMI= 37.3±0.3 kg/m^2^ HbA1c= 7.9±0.1 %	12 months	VLCKD *vs* low-calorie diet	No measure	**↓** HbA1c **↓**Triglycerides **↓**Total chol. **↓** LDL chol. ↑ HDL chol.	No adverse events reported
Goday A 2016 (82)	N=89 Age=54.5±8.4 yr BMI=33.1±1.6 kg/m2 HbA1c=6.9±1.1 %^2^	4 months	Very low-calorie ketogenic diet *vs* low-calorie diet	Blood β-OHB(1.15±0.96mM)	**↓**Triglycerides ↓HbA1c ↓HOMA-IR **↓**body weight **↓**Waist circumference	↑ asthenia ↑ headache ↑ nausea ↑ vomiting ↑ constipation ↑ orthostatic hypotension
Saslow LR 2017 (83)	N=34 BMI=35.9±3.3 kg/m^2^	12 months	VLCKD*vs*MCCR	Blood β-OHB(values between 0.5-3.0mM)	**↓**HbA1c **↓**body weight **↓**Diabetes medications	No adverse events reported
Myette-Côté E 2018 (84)	N=16 Age=64±8 yr BMI=34±8 kg/m2 HbA1c=7±1 %	4 days	VLCKD *vs* low-fat low-glycemic index	No measure	**↔** weight loss **↓** Mean glucose **↓** Fasting glucose	No adverse events reported

BMI, body mass index; T2D, type 2 diabetes; β-OHB, β-hydroxybutyrate; VLCKD, very low carbohydrate ketogenic diet; HOMA-IR, Homeostasis Model Assessment for Insulin Resistance; MCCR, moderate-carbohydrate calorie-restricted. ↓, decrease; ↑, increase; ↔ no changes.

We searched in Medline (PubMed) using the terms “clinical trials” in adults using the query “ketogenic diet” OR “very low carbohydrate ketogenic diet” AND “obesity”, “diabetes”, “NAFLD”, “PCOS”.

## Effects of ketogenic diets in obese subjects

Obesity is recognized as one of the major public health hazards worldwide as it is associated with cardiometabolic and cerebrovascular diseases leading to reduced life quality and increased mortality (85). Over the past decades, there has been a rising prevalence of obesity and its prevention and treatment are mainly based on lifestyle changes, including physical activity and healthy eating habits (86).

Several studies provided robust evidence of the beneficial effect of KDs in reducing body weight in obesity. Importantly, KD-induced weight loss is generally higher than that achieved with an isocaloric non-ketogenic control diet (**Table 2**), as shown in several populations including hyperinsulinemic (48) overweight females (75), overweight/obese subjects (34, 72–74, 76, 77) and in morbidly obese subjects candidate to bariatric surgery (79). In addition, weight reduction following a KD is demonstrated to be accompanied by favourable changes in body composition with a higher decrease in waist circumference (48, 75, 76) and fat mass loss (72, 74), also in subjects who underwent gastric balloon procedure (80), *vs* control diets.

Results on weight management with KDs are generally obtained in animal models (87–90). Mice maintained on a KD exhibited higher weight reduction compared to chow-fed animals both at 6 and 12 weeks. Nevertheless, a few studies in obese mice (*ob/ob*) reported no differences in body weight loss between KD- and chow-fed animals (91, 92).

Favourable data of KD on metabolism have been confirmed also in human studies. A 12-week low-carbohydrate KD intervention led to a more marked improvement in glucose (48, 74, 78) and insulin (48, 49) levels compared to an isocaloric control diet in overweight/obese females (48). The amelioration in metabolic profile associated to a KD in obese subjects, has likely to be ascribed to the very low carbohydrate intake and the consequent decrease in plasma glucose/insulin levels.

KDs might display additional weight loss-independent benefits, particularly on lipid metabolism and inflammatory processes *via* GPCR signaling activation.

Briefly, as already described, GPR109A engagement by β-OHB induces a decrease in adipocyte lipolysis, by blunting hormone sensitive lipase activation (46, 51, 57). This antilipolytic effect in hepatocytes decreases FFA serum levels with a consequent shortage in substrate availability for triglyceride synthesis. Moreover, GPR43 engagement by acetoacetate promotes lipid utilization by increasing plasma LPL activation (56), thus contributing to lipid profile amelioration. In addition to the above-described mechanisms, insulin reduction secondary to carbohydrate restriction might further contribute to a more favourable anti-atherogenic lipid profile by inhibiting HMG-CoA reductase enzyme and consequently endogenous cholesterol biosynthesis (93).

Accordingly, some clinical evidence showed an increase in HDL-cholesterol and a reduction in triglyceride levels in overweight/obese women (48) and in subjects with obesity and dyslipidaemia (77) randomised to a VLCKD compared to those in the isocaloric control diet. In the latter study, although the KD did not affect total low-density lipoprotein (LDL)-cholesterol, it led to a shift from small, dense LDL to large, buoyant LDL, associated to an anti-atherogenic lipid profile (94). In line, a recent study showed that a 2-week low-calorie ketogenic nutrition drink in obese adults was more effective in ameliorating lipid profile compared to an isocaloric balanced nutrition drink (32). Although these favourable mechanistical premises, contrasting literature is present as neutral findings on KD effects on lipid profile were also reported in human studies (74, 75). A very recent systematic review of trials comparing KDs *vs* balanced diets in obese subjects reported no statistically significant differences in total, high-density lipoprotein (HDL) and LDL-cholesterol or triglycerides (95).

These findings may be reconciled taking into consideration the high (saturated) fat intake of these diets which might blunt/abolish the above-mentioned beneficial effects on lipid profile.

Another major point is the possible GPCR-mediated anti-inflammatory effect in course of KD.

A recent study conducted in obese individuals randomised to a 8-week VLCKD *vs* standard diet (both associated to an exercise program) showed a correlation between circulating β-OHB levels and the reduction of visceral fat and inflammatory markers (74) which define the adiposity state, pointing to a direct role of ketones in the amelioration of *metaflammation* (72). These effects should be ascribed to the activation of the β-OHB-GPR109A axis which triggers an anti-inflammatory macrophage phenotype in several animal models (50, 52, 53), ultimately reducing cardiovascular risk.

Thus, KDs might be beneficial in blunting chronic low-grade inflammation which plays a pivotal role in the pathogenesis of cardiometabolic disorders, such as obesity. Nevertheless, future studies are warranted to better explore the impact of this anti-inflammatory mechanism in mediating the beneficial effects of KDs in obesity. Clinical trials on the effects of KDs in subjects with overweight/obesity are reported in **Table 2**, including adverse events.

## Effects of ketogenic diets in individuals with type 2 diabetes

Type 2 diabetes mellitus (T2D) is a major public health global emergency (96). The dietary approach, combined with physical exercise, represents the first-line treatment in T2D. KDs have been taken into consideration, also in recent guidelines (97), among the strategies to adopt in order to obtain weight reduction, ameliorate glucose control and limit the need for anti-hyperglycemic medications in T2D (98).

In KD-fed diabetic rodents, KD strongly reduced blood glucose levels (99–103), improving glucose tolerance (102, 104) and insulin sensitivity (100, 102–104) compared to those fed with a control diet, even at weight loss equipoise (99, 104).

Accordingly, several studies demonstrated KD-associated beneficial effects on weight loss, lipid and glucose parameters, and insulin sensitivity in individuals with T2D (28, 81–84).

Subjects with T2D randomized to a very low-calorie KD for 4 months showed a significant reduction in weight, waist circumference, HbA1c and homeostatic model assessment for insulin resistance (HOMA-IR) compared to those allocated to a standard low-calorie diet (82). Similarly, T2D subjects following a VLCKD showed a decrease in HbA1c levels (81, 83) and an amelioration in lipid profile (81) compared to those on a control diet. A KD-induced decrease in glucose parameters was also confirmed in a short-term (4 days) clinical study (84). Of note, VLCKD consumption was also associated to a reduction in diabetes medication use compared to non-ketogenic control diet (83).

Mechanistically, lipid profile amelioration in T2D by VLCKD could be ascribed to the above-described actions of acetoacetate *via* GPR43 (56) and of β-OHB *via* GPR109A on lipolysis (46, 51) and to the insulin resistance amelioration.

Conversely, the improvement in glucose profile associated to the consumption of a KD in T2D is unlikely to be mediated by GPCR activation, but rather by the limited carbohydrate intake.

Results from controlled clinical trials on the effects of KD in subjects with T2D are summarized in **Table 3**.

Despite these limited but encouraging results, it should be taken into consideration that carbohydrate restriction associated to KDs may increase the risk of hypoglycemia in these patients, especially in those treated with insulin and/or insulin secretagogues. Thus, KDs should be initiated under strict medical supervision, particularly in course of diuretic therapy, in order to avoid hypoglycemia and dehydration (105, 106). Importantly, KDs should not be prescribed to patients treated with sodium-glucose cotransporter (SGLT)-2 inhibitors for their significantly higher risk of developing euglycemic diabetic ketoacidosis (28, 107). Indeed, this drug class enhances the production of KB in the liver, by increasing glucagon levels and reducing the insulin:glucagon ratio *per se* (108–110).

## Effects of ketogenic diets in subjects with type 1 diabetes

Clinical trials investigating KD effects in individuals with type 1 diabetes (T1D) are very limited. The randomization to a VLCKD in a cross-over study reduced glucose variability compared to an isocaloric high-carbohydrate diet (111). Nevertheless, in an uncontrolled study the consumption of a VLCKD was associated to a high risk of dyslipidaemia and to a high frequency of hypoglycaemic episodes in T1D subjects (112). Interestingly, a case report showed that a healthy young woman, in course of a KD for weight reduction, developed diabetic ketoacidosis which was considered the “precipitating factor” leading to T1D diagnosis (113).

Thus, a KD might be appropriate for selected T1D patients, but only following a careful evaluation of the burden of risks (dyslipidemia, diabetic ketoacidosis and hypoglycemia) and benefits (weight loss and better glycemic control) (114). Nonetheless, the latest Standards of Medical Care by American Diabetes Association do not recommend KDs in the medical nutrition therapy for T1D (115).

## Effects of ketogenic diets in individuals with NAFLD

NAFLD is a metabolic liver disease strongly associated with obesity and T2D; its prevalence is increasing - it affects a quarter of global population- along with that of other metabolic disorders. NAFLD is the commonest cause of chronic liver disease and it is defined as an increase in liver fat content, in the absence of secondary cause of steatosis (116). In fact, the clinical onset of the disease is a much more complex process, closely related to insulin resistance and to the limited expandability and dysfunctionality of adipose tissue (117).

In obese mice, a 3-week VLCKD regimen increased pro-inflammatory M1/M2 macrophage ratio in adipose tissue without ameliorating NAFLD, in contrast to the low-fat control diet (118). In line, long-term administration of a KD in mice induced systemic glucose intolerance, hepatic ER stress, steatosis, cellular injury and macrophage accumulation (90). Though, data from the literature are discordant as a very recent paper demonstrated that KD prevented steatosis and insulin resistance by reducing lipogenesis, diacylglycerol accumulation and protein kinase C activity in rat liver (119).

In humans, a very recent randomized 6-week intervention trial showed that the consumption of a KD or an isocaloric low-fat diet similarly and safely reduced liver fat in individuals with NAFLD (120). Nevertheless, randomized trials on subjects with NAFLD are deficient, and the efficacy of KD in NAFLD is still a matter of debate.

KDs may display a dual effect in NAFLD treatment. If on the one hand KD-mediated reduction in insulinemia and lipogenesis and increase in FFA oxidation (121) are beneficial; on the other, the high fat content of the diet might worsen liver fat accumulation.

Therefore, long-term randomized clinical trials are needed to assess safety and efficacy of KDs in NAFLD (122).

## Effects of ketogenic diets in women with polycystic ovary syndrome

PCOS is an endocrine disorder characterized by anovulation, polycystic ovaries and hirsutism. PCOS is often associated to insulin resistance, dyslipidaemia, NAFLD, obesity, and ultimately to an augmented risk for the development of cardiometabolic diseases. To the best of our knowledge, no studies to date have compared the effects of a KD *versus* a non-ketogenic control diet in women with PCOS. Nevertheless, some pilot short-term uncontrolled studies in overweight (123) and obese (124, 125) women with PCOS showed beneficial effects of KDs in reducing body weight, fat mass (123, 124), visceral adipose tissue (123) and cholesterol levels, accompanied by an improvement in insulin sensitivity (reduced HOMA-IR) (123, 124) and to a reduction in the luteinizing hormone (LH)/follicle-stimulating hormone (FSH) ratio and testosterone levels (123–125).

In a very recent study conducted in 18 obese women with PCOS and liver dysfunction the intake of a KD for 12 weeks was more effective compared to a conventional pharmacological treatment (polyene phosphatidylcholine) in reducing fasting glucose, body weight and liver enzymes; both interventions equally decreased plasma estradiol and progesterone levels (126). In conclusion, comparative studies are needed to ascertain whether KDs may display additional endocrinological and metabolic benefits in PCOS, independently of weight loss.

## Discussion

Nutritional ketosis induced through a ketogenic diet leads to higher, but controlled, plasma ketone concentrations (>0.5-1.0 mM). Most human studies provide evidence of a higher effect in short-term weight loss of the KDs compared to isocaloric balanced diets in T2D and obese/overweight subjects, nevertheless controversies remain about the use of KDs in other metabolic disease such as T1D and NAFLD. Scarce evidence exists regarding the effects of KDs in PCOS.

The important weight loss effect with KDs compared to standard diets is largely attributable to higher plasma ketones (31, 127); however, the exact threshold of ketosis needed to control appetite, as well as the exact mechanisms underlying this effect have yet to be established.

KDs might also display additional cardiometabolic benefits, beyond (and in addition to) weight loss, which may rely on different mechanisms. The very limited carbohydrate assumption mainly accounts for the improvement in glucose levels and insulin resistance, often reported in subjects with obesity/diabetes.

Recent evidence supports a different mechanism for KD effects on the amelioration in lipid profile (in particular triglycerides) and inflammation which might be of particular relevance in insulin resistance-related disorders. Ketones can bind specific GPCRs - i.e. GPR109A (50), GPR41 (55) and GPR43 (56) - through which they are able to directly modulate lipid metabolism and inflammation. The activation of acetoacetate-GPR43 axis promotes lipid utilization by plasma LPL activation (56), whilst that of GPR109A by β-OHB decreased adipocytic lipase activity and consequent hepatic triglyceride synthesis (46, 51). In addition, the activation of GPR41 by β-OHB may probably negatively regulate energy intake and metabolism through the suppression of sympathetic activation (55). Comparably, GPR109A-mediated inhibition of NLRP3 inflammasome by β-OHB presumably accounts for the anti-inflammatory effects associated to a KD regimen, leading to an anti-atherogenic polarization of macrophages in M2 phenotype (1, 45, 50, 52).

However, despite a strong preclinical and animal evidence, (45, 50, 52) points to a role of ketones in the reduction of inflammation - which is a common trait of metabolic diseases - a robust clinical confirmation is still lacking. Mechanistic human studies are warranted to ascertain whether GPCR signalling activation by ketones might be of clinical relevance in favourably affecting lipid and inflammatory profiles, independently or in addition to the important weight reduction.

Human intervention studies with KDs are fraught with important limitations since high-quality and long-term evidence is currently scant.

Most interventions span over a period of a few weeks and KD adherence is limited by the very low carbohydrate content (<50g/day) (16). In addition, many studies report only partial data on calories/macronutrients composition of the diets and on the achieved plasma ketone levels, which may account for result heterogeneity among different studies. Ultimately, data on long-term beneficial effects and safety of KD on metabolic/endocrinological disorders are still needed.

In conclusion, although KDs hold a strong potential in the treatment of endocrinological and metabolic disorders due to a broader spectrum of short-term beneficial therapeutic effects in reducing weight and appetite, in ameliorating lipid and glucose profile and the inflammatory milieu, definitive conclusions are difficult to be drawn.

## Author contributions

VS, GC and ADC concepted and designed the review and wrote the manuscript. NTI, GF, GM, MM and VP contributed to the review and interpretation of the literature and drafted the manuscript. ADC critically revised the manuscript. All authors contributed to the article and approved the submitted version.

## Funding

This paper has been supported by “FIL funds for research” from University of Parma to ADC.

## Conflict of interest

The authors declare that the research was conducted in the absence of any commercial or financial relationships that could be construed as a potential conflict of interest.

## Publisher’s note

All claims expressed in this article are solely those of the authors and do not necessarily represent those of their affiliated organizations, or those of the publisher, the editors and the reviewers. Any product that may be evaluated in this article, or claim that may be made by its manufacturer, is not guaranteed or endorsed by the publisher.
